# Health Information Sources Influencing Health Literacy in Different Social Contexts across Age Groups in Northern Thailand Citizens

**DOI:** 10.3390/ijerph19106051

**Published:** 2022-05-16

**Authors:** Nida Buawangpong, Wachiranun Sirikul, Chanya Anukhro, Mathuramat Seesen, Aroon La-up, Penprapa Siviroj

**Affiliations:** 1 Department of Family Medicine, Faculty of Medicine, Chiang Mai University, Chiang Mai 50200, Thailand; nida.buawangpong@cmu.ac.th; 2 Department of Community Medicine, Faculty of Medicine, Chiang Mai University, Chiang Mai 50200, Thailand; wachiranun.sir@cmu.ac.th (W.S.); mathuramat.s@cmu.ac.th (M.S.); 3 Regional Health Promotion Center 1, Department of Health, Ministry of Public Health, Chiang Mai 50100, Thailand; chanya.a@anamai.mail.go.th; 4 Department of Community Public Health, School of Public Health, Walailak University, Nakhon Si Thammarat 80160, Thailand; aroon.la@wu.ac.th

**Keywords:** health literacy, social contexts across age groups, source of health information, Thailand

## Abstract

Health literacy is an essential social determinant of health and has been associated with positive health outcomes. While many sources of health information are widely available, people of different ages also have diverse social contexts and preferences for health information sources. This study aimed to identify health information sources and socio-demographics influencing health literacy in different social contexts across age groups: 15–29 years (young), 30–59 years (middle-age), and ≥60 years (elderly). We obtained secondary data from a cross-sectional study in northern Thailand from March to August 2019. Multivariate linear regression with age group subgroup analysis was used to determine factors associated with general health literacy by the Thai version of the health literacy questionnaire. Receiving health information from physicians was significantly associated with higher general health literacy in young (β 6.45, 95%CI 0.44–12.45; *p* = 0.035) and elderly (β 5.18, 95%CI 1.84–8.52; *p* = 0.002) groups, while receiving health information from health volunteers was significantly associated with a higher general health literacy in the middle-aged (β 2.89, 95%CI 0.38–5.39; *p* = 0.024) group. Although younger participants showed more frequent access to health information via the media than the other age groups, there were no media sources significantly linked with their general health literacy. Health information from physicians is a vital source of health information.

## 1. Introduction

Health literacy is defined as the cognitive and social skills that demonstrate a person’s motivation and ability to find, understand, and appropriately use health information. It is a social determinant of health [1]. Many meanings of health literacy have been provided in previous reports [2]. In 1970, when health education was introduced as a policy in Thailand, health literacy was the first contribution to describing how health information impacts the health care system [3]. Thus, health literacy is shown to be an indicator of health education. Proper health literacy is essential for healthy behavior; for example, successful health information seeking, appropriate dietary consumption, and physical activity can improve health [4]. Indeed, a recent meta-analysis found that higher literacy is associated with a higher uptake of cancer screening programs [5]. Additionally, adequate health literacy is associated with a decreased rate of non-communicable disease [6]. In contrast, the low health literacy of an individual is associated with poor self-care and health outcomes [7,8,9] and with higher health system costs [10].

Traditionally, individuals required consultation with a physician or other health care personnel in order to obtain health information or to receive an answer to a health-related question. Currently, there are numerous new channels and applications for health information [11,12]. Individuals can more easily access any available health resource. For instance, if a person is willing to learn how to take a medication, he or she can obtain this information with a single click by surfing the internet. Therefore, as part of the role of health care personnel and the system, there is a need to understand how people understand and use the various health information sources to make decisions in order to find effective health information and to improve health literacy [13]. In addition, the enhancement of people’s health will be improved by using a person-centered approach involving personal interest, cultural factors, participation, and mutual goal setting [14].

Several studies have been conducted to determine the level of health literacy and the influencing factors. One-half of the population in global developing countries had inadequate health literacy [15,16,17,18], while nearly half of the population in developed countries had limited health literacy [19]. The mean prevalence of limited health literacy in the Southeast Asian region was 55.3 percent [20]. Data from a national assessment of health literacy in the United States showed that most of the participants (53%) had an intermediate level of health literacy, which varied by gender, race, age, socioeconomic status, and sources of health information [21]. Low health literacy was found to correlate with living in a rural area and having a low educational level [22]. Having adequate health literacy was associated with health information-seeking behavior from a healthcare professional, the internet, or other sources of health information [23]. People in different age groups accessed health information resources in different ways [24,25,26,27]. Health literacy can change over time. Understanding people’s preferences and abilities is crucial for improving their health literacy [28]. Additional research is necessary to determine the strategies necessary to increase health literacy [29,30]. To provide tailored, effective health information, it is necessary to identify the types of health information sources that are accessed, used, and are effective. This study aimed to identify the types of health information sources that are used by three age groups, young (15–29 years), middle-aged (30–59 years), and elderly (≥60 years), and to explore the independent association between general health literacy and health information sources, as well as the socio-demographics in northern Thai citizens.

## 2. Materials and Methods

### 2.1. Study Design and Sampling Method

This study used secondary data from a cross-sectional survey of northern Thai citizens from March to August 2019. The derived data were utilized under the authorization of the Regional Health Promotion Center 1 and the Department of Health, Ministry of Public Health, Thailand. The THLS aimed to investigate health literacy and health information sources among Thais aged over 15 years of age. The participants for this project were recruited using an area-stratified, three-stage sampling method to ensure a proportionate distribution from each representative area. Following the THLS procedure, the participants were randomly sampled in three stages: (1) division of the sampling area into non-overlapping strata and random selection of three provinces from the strata of northern Thailand (Chiang Mai, Phrae, and Phayao): (2) random selection of the designated number of 46 enumeration areas (EA) and (3) random selection of the designated number of households from each EA (15 households/each EA; total = 690 households/1736 residents).

### 2.2. Participants and Data Collection Procedure

The study included residents aged 15 years or older who had lived in the sampled households for at least six months before the survey. The sampled households did not include dormitories, prisons, and temples. This study excluded individuals who were unable to communicate or understand the questions due to physical or mental problems. The field investigators contacted the participants in the sampled household for interviews and administered the questionnaire. Participants were invited for face-to-face interviews with investigators who were healthcare workers at the DoH. They were trained in health literacy assessment, the survey method, interviews, and data collection by the principal investigators from the DoH and the Health Intervention and Technology Assessment Program (HITAP), Ministry of Public Health of Thailand. They were also supervised during the survey period by DoH area supervisors. The field investigators could exclude the participants who met the exclusion criteria or consult the area supervisors if unsure. If any member of the sampled household was not present on the interview date, field investigators returned to interview no more than three times. All participants had given informed consent before the interview.

A standard protocol was provided by the investigators from the DoH, HITAP, and National Statistics Office (NSO) to ensure standardization and quality assurance in the data collection. The field investigators and the supervisors from each area of the study evaluated the quality of the collected data: completeness, consistency, sample characteristics, and data correction. The area supervisors also provided weekly technical reports and sent the data to the principal investigator team.

**Figure 1:**
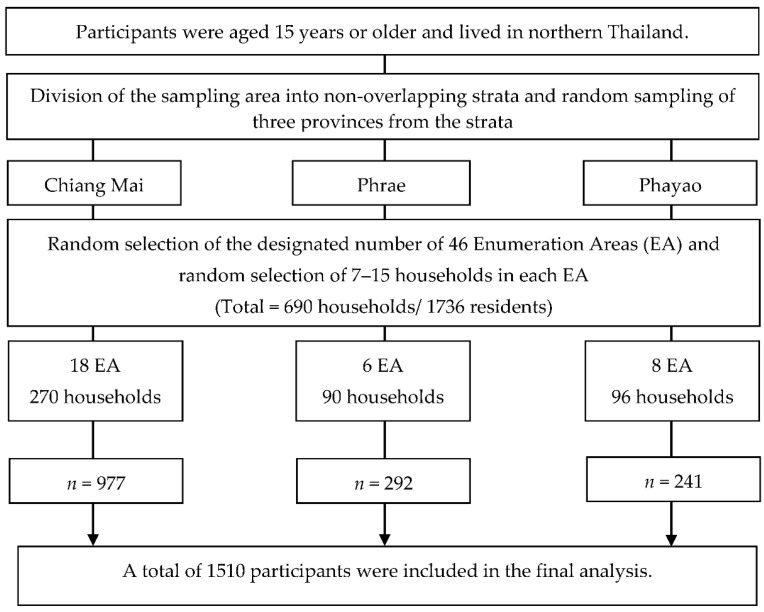
Flow diagram of the recruitment of sampled participation into this study.

After excluding participants who met the exclusion criteria, the total number of study participants was 1510 (87.0%) of 1736 expected residents (Figure 1); there were no missing data or unsatisfactory responses. Of 1510 participants, 214 (14.2%) were between 15 and 29 years old (young), 691 (45.8%) were between 30 and 59 years old (middle-age), and 605 (40.0%) were aged over 60 years (elderly).

### 2.3. Variables and Measurement

#### 2.3.1. Participant Characteristics, Socio-Demographics, and Health Information Sources

A questionnaire was used to collect data on demographic and health characteristics and included sex, age, marital status, education, occupation, self-perception of income (insufficient, sufficient, and more than sufficient), number of household members, living location (in/outside municipality), and the number of household members and history of diagnosed chronic diseases (hypertension, diabetes, dyslipidemia, heart disease, stroke, asthma, chronic obstructive pulmonary disease, cancer, and osteoporosis). Other information concerned the sources of health information and comprised of two types: (1) media such as television, commercial and public radio, community radio (community announcement via loudspeakers/radio broadcasting by community leaders or members), magazines, and from the internet. The internet comprised websites, search engines, instant messaging (e.g., the LINE application, (a secure messaging system similar to Facebook Messenger), and social media platforms (Facebook, etc.); (2) professional/personal contacts such as physicians, nurses, pharmacists, dentists, public health workers, health volunteers, and family and friends.

#### 2.3.2. The Thai Version of the Health Literacy Questionnaire (THLQ)

The Thai version of the health literacy questionnaire (THLQ) was developed by the THLS investigators, who were public health experts from the DoH and HITAP. It was modified from the European Health Literacy Survey Questionnaire (HLS-EU-Q) [2,31]. The THLQ contained 42 items and used self-reporting to measure health literacy. It had five subindices of health information relevant to decision-making in four topics: health services, disease prevention, health promotion, and consumer protection, as shown in Table 1. Each item was rated on a 4-point Likert scale from cannot do to very easy (0 = never or cannot do, 1 = very difficult, 2 = difficult, 3 = easy, 4 = very easy). The possible scores on the THLQ for general health literacy ranged from 0 to 168. This questionnaire was validated in 240 Thais aged 15 and older from a cross-sectional study conducted between 1 and 15 December 2018 in Bangkok and Chonburi provinces in the central region of Thailand. The Cronbach’s coefficient alpha for the reliability test of general health literacy scales was 0.941. The reliability test of the five THLQ subindices obtained a Cronbach’s coefficient alpha of between 0.821 and 0.902. The reliability tests for general and subindices of health literacy in the validation study and this study are shown in Table S1.

### 2.4. Statistical Analysis

All of the statistical analyses were conducted using the STATA statistical software program (Stata Corp. 2019, Stata Statistical Software: Release 16, Stata Corp LLC, College Station, TX, USA). The participants’ characteristics, sources of health information, and health literacy scores were described by a frequency with a percentage for the categorical data, a mean with a standard deviation (SD) for the parametric data, and a median with an interquartile range (IQR) for the non-parametric data. The chi-square test was used to examine a statistical difference in the proportion of participants’ characteristics and sources of health information among age groups: 15–29 years (young), 30–59 years (middle-age), and over 60 years (elderly). Age groups were generated based on diversity across social contexts, health risks, and potential effects of health information from various sources: adolescents and young adults (aged 15–29 years), middle-aged individuals (aged 30–59 years), and older or retired individuals (aged ≥60 years) [32,33,34]. The health literacy scores and subindices were compared between the participants in the different age groups as defined, using a one-way ANOVA with a post-hoc Scheffe multiple-comparison test for parametric data and a Kruskal–Wallis test with a post-hoc Tukey multiple-comparison test for non-parametric data. The full exploratory model using a multivariate linear regression was analyzed to explore the association between sources of health information and general health literacy by 42 items of the THLQ with pre-defined potential associated factors including sex, age, marital status, education, occupation, income, household size, and living location. Group analysis was performed to determine the potential sources of health information that were associated with general health literacy in the different age groups. The results of this study were reported according to the strengthening of the reporting of observational studies in the Epidemiology (STROBE) checklist. All statistical analyses were two-sided, and a *p*-value ≤ 0.05 was considered statistically significant.

### 2.5. Ethical Considerations

This study was conducted following the Declaration of Helsinki, and the protocol was approved by the Research Ethics Committee, Faculty of Medicine, Chiang Mai University, Thailand (Study code: COM-2564-08405).

## 3. Results

### 3.1. The Study Participants’ Characteristics

The participants’ response rate was 87.0% (1510/1736). The details of the participants’ characteristics are shown in Table 2. The proportion of females in the different age groups was not significantly different. The proportions of education levels, marital status, and occupations in the different age groups were significantly different. Most elderly participants were married (69.8%) or divorced or widowed (24.1%). The most common marital status in the middle-aged group was married (72.8%). In the young group, 73.4% were single. In the elderly group, the most common education level was the primary school level (69.8%), followed by illiteracy (15.4%). Most participants in the middle-aged group had a primary school (40.4%) or a high school/vocational school education (31.3%), while the young age group had an education level of high school/vocational school (67.7%) or university (24.3%). The majority of the elderly participants were retired or unemployed (49.4%). Half of the middle-age worked as farmers or laborers (50.5%), and most of the young participants were students (42.5%). The number of underlying diseases was significantly different among the different age groups as well as household sizes as defined by the number of members. The majority of the participants (62.4%) lived in the municipality and reported having an insufficient income (49.8%) or a sufficient income (41.5%). There were no statistically significant differences in self-perception of income and living location among the age groups.

### 3.2. Sources of Health Information

**Figure 2:**
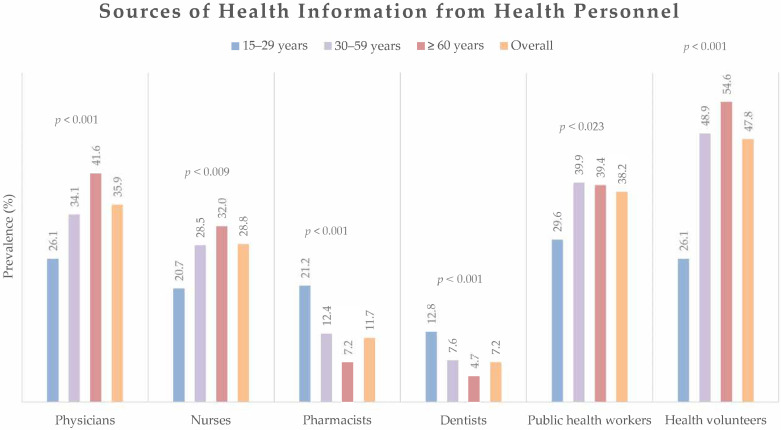
Sources of health information obtained from health personnel by participants overall and by age group. The differences in prevalence of received health information from health personnel among age-groups were analyzed by chi-square test.

From Figure 2, the participants reported that the highest proportion of information sources from health personnel was health volunteers (47.8%), followed by public health workers (38.2%) and physicians (35.9%). The proportion of health information obtained from health personnel varied significantly among the different age groups. However, the common sources of health information remained health volunteers, public health workers, and physicians in all age groups.

**Figure 3:**
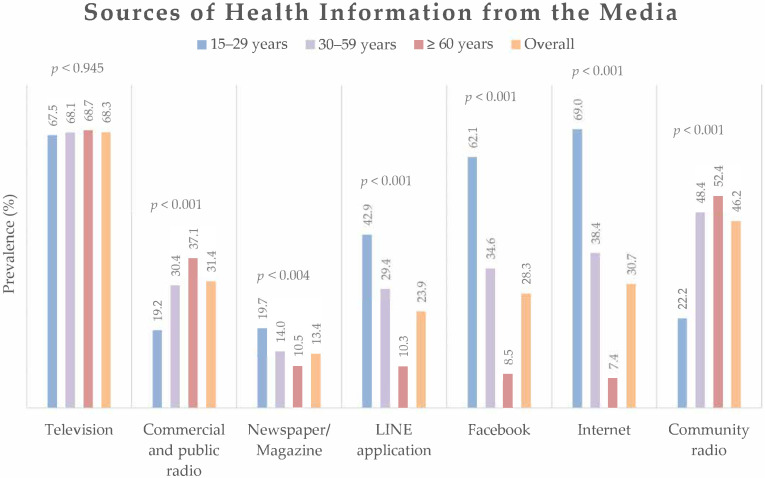
Sources of health information obtained from media overall and by age group. The differences in prevalence of received health information from the media among age-groups were analyzed by chi-square test.

As illustrated in Figure 3, the common source of health information from the media was television, which was not significantly different across the different age groups. The younger participants reported more frequently accessing health information via digital platforms, particularly internet browsers (69.0%), Facebook (62.1%), and the LINE applications (42.9%). In contrast, middle-aged and elderly participants received health information mostly from other traditional media, particularly from community radio and commercial and public radio.

### 3.3. General and Subindices Health Literacy

**Figure 4:**
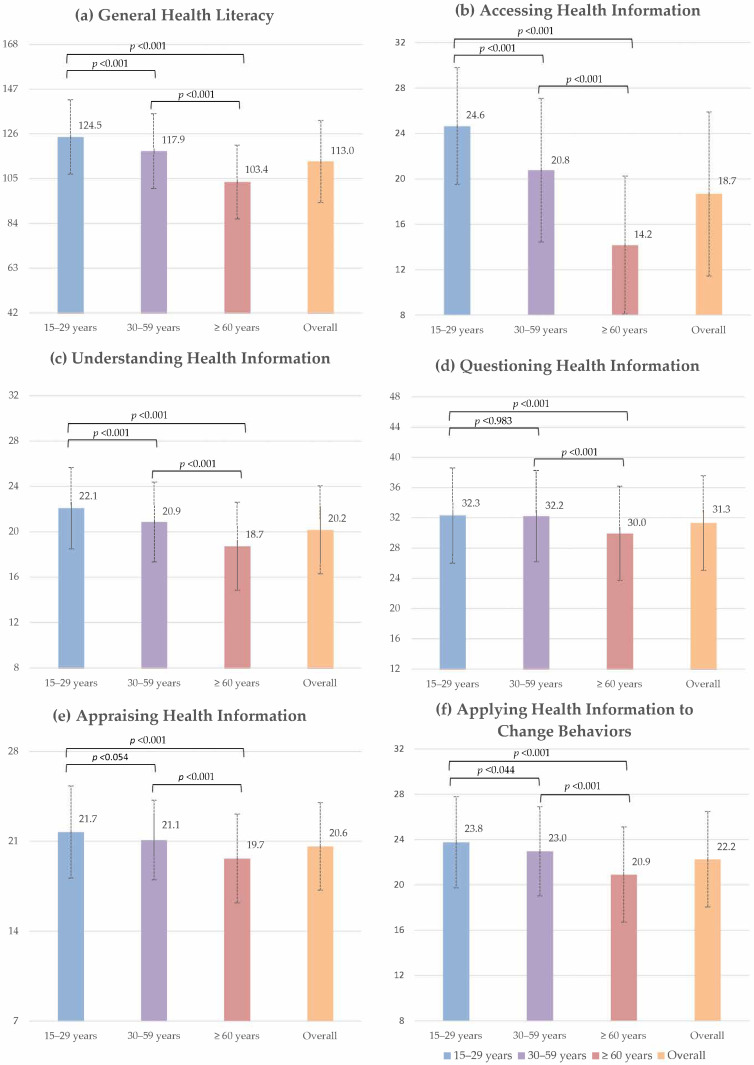
Bar plots illustrating the mean and standard deviation of general and subindices of health literacy stratified by age-groups.

Figure 4 illustrates the general and subindex health literacy scores stratified by the age groups. The mean general health literacy score was 113.03 (SD ± 19.24). In comparison to the other age groups, young participants showed the highest mean general and subindex health literacy scores, whereas the elderly had the lowest mean general and subindex health literacy scores. Between young and middle-aged participants, only the mean subindex scores for questioning and appraising health information were not statistically different, while the other health literacy scores of young participants were significantly higher than the middle-aged group. The additional data on the general and subindex health literacy scores are presented in the supplementary data (Table S2).

### 3.4. Exploration of the Factors Associated with General Health Literacy

Table 3 shows the results from the full exploratory model with analysis by age group using multivariate linear regression to determine the factors associated with general health literacy. Participants aged 60 and older had lower general health literacy (β −9.26, 95%CI −12.62 to −5.89; *p* < 0.001) when compared to the young group. Female gender (β 1.67, 95%CI 0.06 to 3.28; *p* = 0.042) and divorced or widowed (β −4.43, 95%CI −7.55 to −1.31; *p* = 0.005) were both significantly associated with differences in general health literacy in the full model. Higher education levels compared to illiteracy and employment also showed a significant association with higher general health literacy, while the other characteristics were not significantly associated. Physicians, health volunteers, the internet, LINE application, magazines or newspapers, and commercial radio were all significantly associated with higher general health literacy, whereas television, the most commonly accessed source of information in all age groups, was not related to health literacy. Furthermore, receiving health information from community radio (β −2.87, 95% CI −4.57 to −1.18; *p* = 0.001) had a negative association with general health literacy.

The subgroup analysis of age groups is shown in Table 4. There was no significant association between the participant characteristics and general health literacy in the young group. Higher education levels above illiteracy remained significantly related to increased general health literacy in the groups of middle-aged and elderly participants. However, only employed elderly participants were associated with higher general health literacy. Receiving health information from physicians remained associated with substantially higher general health literacy in the young (β 6.45, 95%CI 0.44 to 12.45; *p* = 0.035) and elderly (β 5.18, 95%CI 1.84 to 8.52; *p* = 0.002) groups while receiving health information from health volunteers was only associated with a significant increase in the general health literacy of the middle-aged group (β 2.89, 95%CI 0.38 to 5.39; *p* = 0.024). In the young group, there were no sources of health information from the media that were associated with general health literacy. Surfing for health information via the internet was significantly associated with higher general health literacy in middle-aged participants (β 5.41, 95%CI 2.27 to 8.54; *p* < 0.001). For the elderly group, receiving health information from traditional media, including radio (β 4.15, 95%CI 1.44 to 6.85; *p* = 0.003) and magazines or newspapers (β 5.97, 95%CI 1.89 to 10.06; *p* = 0.004), was significantly associated with greater general health literacy, whereas receiving health information from community radio (β −3.13, 95%CI −5.71 to −0.55; *p* = 0.018) showed a negative association with general health literacy.

## 4. Discussion

Numerous health literacy resources are available and accessible worldwide. As a result, it is essential to validate the reliability of health information. While there are numerous sources of health information, only meaningful and effective sources can increase people’s health literacy, resulting in a better health outcome. This research established that the health information obtained from physicians, health volunteers, and most media sources were significantly associated with higher health literacy, but community radio was significantly related to lower health literacy for the general population. However, our study demonstrated that the association of different health information sources with health literacy varied by age group. Higher general health literacy was significantly correlated with receiving health information from physicians in the young and elderly age groups and receiving health information from health volunteers in the middle-aged group. Although young participants showed more frequent access to health information via the media than the other age groups, there were no media sources that were significantly linked with their general health literacy. Both the internet and magazines or newspapers, which are common information sources, had a significant association with the middle-aged group’s health literacy. Elderly participants who received health information through the traditional media, including radio and magazines or newspapers, also showed a significantly higher general health literacy. Interestingly, the negative association between receiving health information from community radio and elderly health literacy remained significant.

### 4.1. Sociodemographic Factors Influencing Health Literacy

The current study illustrated that higher levels of health literacy were associated with younger age, female gender, higher educational levels, and recent employment. The relationship between aging and reduced health literacy could be influenced by reduced independence, social skills, and the media used by the elderly [35,36]. However, the studies on the influence of gender on health literacy are still unclear, and some studies have reported different results [37,38,39]. Additionally, people’s health literacy affects decision-making and behavior in everyday life. Health literacy is a social determinant of health, and it is also associated with other life factors: education and type of job [40]. Low education results in a poor understanding of health information and, consequently, poor decision-making regarding health matters [41,42,43].

### 4.2. Sources of Health Information

Numerous sources of health information from health personnel have been identified in this study. Access to health information from healthcare professionals has become easier in recent years. Not only when individuals visit hospitals, clinics, or drug stores but also through direct communication, such as telemedicine, text messaging, or video conferencing via smartphone or tablet applications [44,45,46]. In addition, Thailand’s National Health Service developed a primary care system to maintain a high standard of patient care. A proactive care process is in place. Individuals in each service area can receive health care from a multidisciplinary team comprised of physicians, nurses, physical therapists, pharmacists, and others that improve health outcomes [47,48]. Health volunteers are also employed to improve population health outcomes. Health volunteers are individuals in each health area who volunteer to assist people in their locality in achieving their goal of improving health outcomes. They perform a variety of health-related tasks, including enhancing health education, screening for diseases, and preventing epidemics [49,50,51]. Due to the fact that they are also residents of the area, they often gain increased community support and access to individuals in their area [52].

The study demonstrated that different sources of health information have different associations with the health literacy of people of various ages. From the group of health personnel, health volunteers were the most accessed, followed by public health workers and physicians, except for the elderly group, where physicians were the second most accessed, followed by public health workers. This may be because elderly people have an increased number of underlying illnesses [53], and the elderly population requires regular visits to physicians. A proactive service system should therefore target this age group to enhance self-care and disease control. Television was the most popular choice for accessing health information from media sources. The young group reported a high prevalence of accessing health information from the internet, Facebook, and the LINE application. The internet and social media have become primary sources of health information for many people in the digital era [54].

### 4.3. Relationship between the Health Information Sources and Health Literacy

The results indicated that health information from physicians and health volunteers was associated with a significantly higher health literacy level. Physicians play a major role in the health care service in developing the guidelines of healthcare and the systems designed to deliver them [55]. Health information from physicians, especially the personal doctor who continually treats the patient, can provide specific advice on health education for each patient. Implementing a health care session for patients when they visit would improve a patient’s health literacy. Another effective proactive health care service is that provided by health volunteers. The development and expansion of the capacity and number of health volunteers are recommended [56,57,58]. Health volunteers can also improve the self-care and health outcomes of the patients by providing health information to enhance awareness and self-efficacy [59].

People not only find information from health personnel but also seek it out themselves. From media sources, the internet, the LINE application, radio, and magazines or newspapers demonstrated a significant positive association with health literacy. Even though the use of both radio and newspapers was lower than others, it still showed an association with higher health literacy. In contrast, television, the most popular source of health information for all age groups, had no significant correlation. The young and middle-aged groups tended to use more social media and digital communication platforms via the internet than the elderly group. People need to take care of their health and deal with health problems; they can do this by increasing the amount of health information they receive [12]. Mass communication, for example, television, radio, and newspapers, is the traditional source of providing health information [60]. Creating health education and improving the quality of health information via this platform may improve people’s health literacy [61]. Another important part of using media sources for providing health information is the way that this information is communicated. The information should be simple, clear, and useful [62]. Social media is increasingly a factor in daily life and has shown a positive effect on health [63,64]. Nevertheless, the reliability and safety of the health information obtained on social media need evaluation [65].

### 4.4. Health Literacy in COVID-19 Pandemic

Health literacy was also affected by the global health situation during the COVID-19 pandemic. Inadequate health literacy is associated with a poor understanding of COVID-19 symptoms and poor behavior to prevent infection [66]. Health literacy was a critical factor in vaccine hesitancy [67]. Health information, for example, guidelines or health education for COVID-19 vaccination, could enhance health literacy and deal with vaccine hesitancy [68]. Governments and healthcare services use social media as a way of sharing health information to reduce transmission and enhance people’s understanding. However, unclear or false health information can lead to misunderstanding. It is very important to provide true, reliable, and accessible health information to the general population [69].

### 4.5. Generalizability, Implications and Future Studies

According to the Thai Health Literacy Survey (THLS), health literacy and sources of health information among the general population in different regions were not significantly different. Our findings could be generalized to the Thai population and others with similar demographics, social contexts, and preferences for sources of health information. However, additional research in other societies remains necessary due to the high degree of diversity in social contexts and available sources of health information across countries. Since we have various sources of health information, they can be a way to improve people’s health literacy. Many health policies could be developed according to the study’s results to improve the population’s level of health literacy. For instance, the public health sector could provide more reliable and validated health information in the mainstream media. Regularly updated health information for health personnel is also suggested, especially for those who are involved in proactive health care. More accessible and reliable health information resources are needed. It would be beneficial to enhance the public’s capacity to assess the quality of health information sources [70,71]. This would improve the population’s health literacy.

Future research could be conducted on the factors affecting health literacy at the health district level. That would help policy formulation and the development of appropriate interventions. Qualitative data in conjunction with quantitative survey results in a specific population, such as cultural, religious, or ethnic groups, will help to clarify the relationship between those factors and health literacy.

### 4.6. Strengths and Limitations

The study’s strength was the large sample size of people from northern Thailand. The survey method was an excellent way to collect a large amount of data from a large number of people. Additionally, it contained data from various ages and social contexts. There were some limitations. First, the results should be interpreted, and implications drawn with caution. Because of the nature of a cross-sectional study, it was not possible to determine the causal direction of the relationship. It is possible that an individual’s health literacy affects the sources of information they use. Second, questionnaires were used to reflect the participants’ perspectives and experiences regarding the level of implementation of health literacy skills. They did not assess the knowledge or ability to perform specific tasks. However, this questionnaire demonstrated high validity for evaluating the health literacy state of the Thai population aged 15 years and older in different social contexts. Finally, the survey’s findings indicated that individuals with insufficient health literacy have difficulty accessing, understanding, and making decisions about health, but the findings cannot be extended to health or behavioral outcomes such as disease control.

## 5. Conclusions

Numerous health information resources are available to people of all ages. Health information from physicians was associated with higher general health literacy in the young and elderly age group, while health information from health volunteers was associated with higher general health literacy in the middle-aged group. Commercial and public radio, magazines or newspapers, and the internet were associated with higher general health literacy in the general population and specific age groups. A negative association between health information from community radio and elderly health literacy was shown. Physician-provided health information and education is the most effective resource for patients seeking advice and improving self-efficacy, health behavior, or disease control. Physicians should incorporate health information counseling into their practice. Other health resources from health care professionals and media should be evaluated to achieve a higher level of health literacy.

## Figures and Tables

**Table 1:** The Thai version of the health literacy questionnaire (THLQ) items.

Subindices	Topics		Questions
(1) Accessing information relevant to health (8 items)	Health services	S1	What to do if someone is unconscious
		S2	Symptoms require immediate hospitalization
	Disease prevention	S3	Health check-up or vaccinations should be given
		S4	Self-prevention from communicable diseases
	Health promotion	S5	How to maintain health
		S6	Proper way to manage stress
	Consumer protection	S7	Reliable information about medicines, cosmetics, herbs, and supplements
		S8	Reliable information about health services, new products, or equipment
(2) Understanding information relevant to health (7 items)	Health services	U9	Public media, online media or what to do in a medical emergency
	Disease prevention	U10	Why you should have a health screening
		U11	Understand why you need a vaccine
	Health promotion	U12	Warnings about how important and necessary healthy behaviors are
		U13	How to maintain good mental health
	Consumer protection	U14	Understand the labels for drugs, cosmetics, dietary supplements, herbs, medical devices, and dangerous substances
		U15	Description of new health services, products, or equipment
(3) Questioning information relevant to health (12 items)	Health services	Q16	Ask your doctor about your rights, expenses, and concerns
		Q17	Ask the nurse about your rights, expenses, and concerns.
		Q18	Ask the hospital worker about your rights, expenses, and concerns.
	Disease prevention	Q19	Ask your doctor about the disease and the practice of prevention
		Q20	Ask the nurse about the disease and the practice of prevention
		Q21	Ask the health volunteer about the disease and the practice of prevention
	Health promotion	Q22	Ask your doctor for information on how to stay healthy
		Q23	Ask the nurse for information on how to stay healthy
		Q24	Ask the health volunteer for information on how to stay healthy
	Consumer protection	Q25	Ask your doctor about health products
		Q26	Ask the nurse about health products
		Q27	Ask the health volunteer about health products
(4) Appraising information relevant to health (7 items)	Health services	D28	Decide how you follow an emergency information
	Disease prevention	D29	Decide how you follow a complete treatment plan
		D30	Decide what you will do in order to use the drug correctly
	Health promotion	D31	Decide how you should exercise
		D32	Decide what foods to buy or how to cook
		D33	Decide what activities you should do to reduce stress
	Consumer protection	D34	Decide to choose a supplement, nutrition, or herbs
(5) Applying information to change behaviors (8 items)	Health promotion	B35	Choose bland food more than sweet, salty, and oily food
	Disease prevention/Health promotion	B36	Do not share a spoon with others while eating
	Health promotion	B37	Exercise to strengthen muscles 3–4 days a week.
	Disease prevention/Health promotion	B38	Manage your stress appropriately
	Consumer protection	B39	Read and review the information provided with cosmetics, supplements, or herbs
		B40	Inspect health products, services, cosmetics, medical devices, and hazardous substances
	Health services	B41	If you find someone drowning, help by “yelling, throwing, handing” and calling 1669.
	Health promotion	B42	Encourage all children to receive developmental assessment and vaccination as needed

**Table 2:** Characteristics of the study participants by age-groups.

Characteristics	Total (*n* = 1510)	Age Group (Years)			*p*-Value
		15–29 (*n* = 214)	30–59 (*n* = 691)	≥60 (*n* = 605)	
	*n* (%)	*n* (%)	*n* (%)	*n* (%)	
Gender					
Male	614 (40.7)	94 (43.9)	279 (40.4)	241 (39.8)	0.566
Female	896 (59.3)	120 (56.1)	412 (59.6)	364 (60.2)	
Marital status					
Single	323 (21.4)	159 (74.3)	127 (18.4)	37 (6.1)	<0.001 **
Married	976 (64.7)	52 (24.3)	502 (72.8)	422 (69.8)	
Divorced/Widowed	210 (13.9)	3 (1.4)	61 (8.8)	146 (24.1)	
Education level					
Illiterate	142 (9.4)	4 (1.9)	45 (6.5)	93 (15.4)	<0.001 **
Primary school	713 (47.2)	13 (6.1)	279 (40.4)	421 (69.6)	
High school/vocational school	423 (28.0)	145 (67.7)	216 (31.3)	62 (10.2)	
University	232 (15.4)	52 (24.3)	151 (21.8)	29 (4.8)	
Occupation					
Unemployed/retired/housekeeper	398 (26.4)	27 (12.6)	72 (10.4)	299 (49.4)	<0.001 **
Official worker	113 (7.5)	21 (9.8)	83 (12.0)	9 (1.5)	
Farmer/laborer	624 (41.3)	54 (25.3)	349 (50.5)	221 (36.5)	
Merchant/business owner	281 (18.6)	21 (9.8)	184 (26.6)	76 (12.6)	
Student	94 (6.2)	91 (42.5)	3 (0.4)	0 (0.0)	
Self-perception of income					
Insufficient	752 (49.8)	109 (50.9)	351 (50.8)	292 (48.3)	0.802
Sufficient	627 (41.5)	84 (39.3)	284 (41.1)	259 (42.8)	
More than sufficient	131 (8.7)	21 (9.8)	56 (8.1)	54 (8.9)	
Household size (person)					
One	140 (9.3)	13 (6.1)	67 (9.7)	60 (9.9)	<0.001 **
Two	619 (41.0)	46 (21.5)	281 (40.7)	292 (48.3)	
Three to four	665 (44.0)	126 (58.9)	311 (45.0)	228 (37.7)	
Five or more	86 (5.7)	29 (13.5)	32 (4.6)	25 (4.1)	
Living location					
In municipality	942 (62.4)	136 (63.6)	410 (59.3)	396 (65.4)	0.071
Outside municipality	568 (37.6)	78 (36.4)	281 (40.7)	209 (34.6)	
Number of chronic conditions					
None	816 (54.0)	195 (91.1)	428 (61.9)	193 (31.9)	<0.001 **
One	363 (24.0)	16 (7.5)	160 (23.2)	187 (30.9)	
Two	214 (14.2)	3 (1.4)	72 (10.4)	139 (23.0)	
Three or more	117 (7.8)	0 (0.0)	31 (4.5)	86 (14.2)	

Chronic conditions include hypertension, diabetes, dyslipidemia, heart disease, stroke, asthma, chronic obstructive pulmonary disease, cancer, and osteoporosis; ** Significant association at *p* < 0.001.

**Table 3:** The exploratory models of the factors associated with general health literacy.

Variables	β	(95%CI)	*p*-Value
Characteristics and socio-demographics			
Age (years)			
15–29	Ref.		
30–59	−2.45	−5.23 to 0.33	0.084
≥60	−9.26	−12.62 to −5.89	<0.001 **
Gender			
Male	Ref.		
Female	1.67	0.06 to 3.28	0.042 *
Marital status			
Single	Ref.		
Married	0.29	−1.99 to 2.58	0.800
Divorced/Widowed	−4.43	−7.55 to −1.31	0.005 *
Education level			
Illiterate	Ref.		
Primary school	14.53	11.52 to 17.55	<0.001 **
High school/vocational school	20.77	17.32 to 24.22	<0.001 **
University	23.66	19.75 to 27.56	<0.001 **
Occupation			
Unemployed	Ref.		
Employed	2.11	0.14 to 4.07	0.036
Self-perception of income			
Insufficient	Ref.		
Sufficient	0.55	−1.08 to 2.18	0.507
More than sufficient	2.69	−0.16 to 5.54	0.064
Living location			
In municipality	Ref.		
Outside municipality	−1.41	−3.14 to 0.32	0.109
Household size (no. of person)	−0.55	−1.61 to 0.51	0.311
No. of chronic conditions	−0.13	−0.93 to 0.68	0.756
Source of health information from health personnel			
Health volunteer	2.64	0.90 to 4.37	0.003 *
Public health worker	0.97	−0.75 to 2.71	0.270
Physician	4.02	1.62 to 6.07	0.001 **
Nurse	0.46	−1.82 to 2.73	0.695
Pharmacist	−0.50	−3.19 to 2.19	0.715
Source of health information from media			
Television	−0.43	−2.20 to 1.33	0.631
Community radio	−2.87	−4.57 to -1.18	0.001 *
Commercial and public radio	1.87	0.06 to 3.68	0.043 *
Internet	3.51	1.08 to 5.93	0.005 *
Facebook	1.21	−1.32 to 3.75	0.348
LINE application	2.63	0.25 to 5.01	0.031 *
Magazine/Newspaper	4.62	2.24 to 7.01	<0.001 **
Constant	88.17	82.41 to 93.92	<0.001 **
R = 0.405, Adjusted R^2^ = 0.394, F = 36.38, *p* < 0.001 **			

The exploratory model by a multiple Gaussian regression analysis; Ref. = reference, reference group including female gender, single, illiteracy, unemployed/housekeeper, poor income, and living outside municipality; * Significant association at *p* < 0.05, ** Significant association at *p* < 0.001.

**Table 4:** Subgroup analysis by age groups of the factors associated with general health literacy.

Variables	Subgroup Analysis by Age Groups								
	15–29 Years (*n* = 214)			30–59 Years (*n* = 691)			≥60 years (*n* = 605)		
	β	(95%CI)	*p*-Value	β	(95%CI)	*p*-Value	β	(95%CI)	*p*-Value
Characteristics and socio-demographics									
Gender									
Male	Ref.			Ref.			Ref.		
Female	1.83	−2.61 to 6.26	0.418	1.36	−1.02 to 3.74	0.262	1.83	−0.84 to 4.49	0.178
Marital status									
Single	Ref.			Ref.			Ref.		
Married	−0.18	−5.48 to 5.11	0.773	−0.49	−3.51 to 2.53	0.751	3.35	−1.96 to 8.66	0.215
Divorced/Widowed	−10.98	−29.26 to 7.30	0.237	−2.60	−7.38 to 2.17	0.285	−2.01	−7.60 to 3.58	0.481
Education level									
Illiterate	Ref.			Ref.			Ref.		
Primary school	−16.43	−35.08 to 2.22	0.084	22.08	17.06 to 27.09	<0.001 **	11.13	7.22 to 15.05	<0.001 **
High school/vocational school	−3.69	−20.68 to 13.31	0.669	27.49	22.20 to 32.77	<0.001 **	16.21	10.78 to 21.63	<0.001 **
University	−0.21	−17.77 to 17.34	0.981	30.44	24.58 to 36.31	<0.001 **	18.89	11.78 to 26.00	<0.001 **
Occupation									
Unemployed	Ref.			Ref.			Ref.		
Employed	−5.59	−12.33 to 1.16	0.104	2.14	−1.57 to 5.84	0.258	3.33	0.80 to 5.86	0.010 *
Self-perception of income									
Insufficient	Ref.			Ref.			Ref.		
Sufficient	−0.45	−5.01 to 4.12	0.848	−0.92	−3.32 to 1.48	0.451	1.99	−0.61 to 4.59	0.133
More than sufficient	7.14	−0.34 to 14.63	0.061	0.28	−4.11 to 4.67	0.899	4.00	−0.40 to 8.41	0.075
Living location									
In municipality	Ref.			Ref.			Ref.		
Outside municipality	1.25	−3.75 to 6.24	0.623	−0.27	−2.82 to 2.27	0.833	−2.11	−4.86 to 0.64	0.133
Household size (no. of person)	−1.59	−4.54 to 1.37	0.291	−0.24	−1.82 to 1.33	0.761	−0.69	−2.37 to 0.99	0.418
No. of chronic conditions	−1.02	−7.32 to 5.28	0.749	−0.15	−1.42 to 1.13	0.822	−0.25	−1.30 to 0.80	0.640
Source of health information from health personnel									
Health volunteer	5.37	−0.83 to 11.57	0.089	2.89	0.38 to 5.39	0.024 *	0.49	−2.16 to 3.14	0.716
Public health worker	−2.59	−8.48 to 3.29	0.386	1.14	−1.47 to 3.75	0.391	1.56	−1.05 to 4.17	0.241
Physician	6.45	0.44 to 12.45	0.035 *	1.74	−1.66 to 5.12	0.315	5.18	1.84 to 8.52	0.002 *
Nurse	0.21	−5.96 to 6.38	0.947	−0.83	−4.48 to 2.82	0.655	1.90	−1.53 to 5.34	0.277
Pharmacist	3.27	−3.10 to 9.63	0.312	−0.42	−4.37 to 3.52	0.832	−1.15	−6.17 to 3.85	0.651
Source of health information from media									
Television	−3.05	−8.03 to 1.93	0.228	−0.69	−3.31 to 1.94	0.609	0.41	−2.37 to 3.18	0.774
Community radio	−4.68	−10.70 to 1.33	0.126	−1.89	−4.32 to 0.54	0.128	−3.13	−5.71 to −0.55	0.018 *
Commercial and public radio	−2.75	−8.74 to 3.24	0.366	0.84	−1.86 to 3.55	0.541	4.15	1.44 to 6.85	0.003 *
Internet	0.45	−5.15 to 6.06	0.874	5.41	2.27 to 8.54	<0.001 *	1.23	−5.01 to 7.48	0.698
Facebook	0.36	−4.71 to 5.45	0.886	0.85	−2.57 to 4.28	0.626	5.03	−1.48 to 11.53	0.130
LINE application	3.12	−1.75 to 8.00	0.208	2.92	−0.36 to 6.20	0.081	2.89	−2.73 to 8.52	0.313
Magazine/Newspaper	4.17	−1.52 to 9.85	0.150	4.43	0.85 to 8.00	0.015 *	5.97	1.89 to 10.06	0.004 *
Constant	129.78	109.45 to 150.10	<0.001 **	86.89	77.94 to 95.84	<0.001 **	85.29	75.80 to 94.78	<0.001 **
	R = 0.240, Adjusted R^2^ = 0.137 F = 2.34, *p*= 0.001 **			R = 0.335, Adjusted R^2^ = 0.309, F = 13.26, *p* < 0.001 **			R = 0.321, Adjusted R^2^ = 0.290, F = 10.41, *p* < 0.001 **		

The exploratory model by a multivariate linear regression analysis; Ref. = reference, reference group including female gender, single, illiteracy, unemployed/housekeeper, poor income, and living outside municipality; * Significant association at *p* < 0.05, ** Significant association at *p* < 0.001.

## Data Availability

The data presented in this study are available on request from the correspondent author.

## Supplementary Materials

The following supporting information can be downloaded at: https://www.mdpi.com/article/10.3390/ijerph19106051/s1, Table S1: The reliability test of five THLQ subindices, Table S2: Frequency and percentage of health literacy levels in the sample population.
