# Supplementary material for: Health Information Sources Influencing Health Literacy in Different Social Contexts across Age Groups in Northern Thailand Citizens
Source: Int J Environ Res Public Health. 2022 May 16;19(10):6051. doi: 10.3390/ijerph19106051 (PMC9141654; doi:10.3390/ijerph19106051)
Supplement: Supplementary file 1 [file ijerph-19-06051-s001.zip › ijerph-1662772-supplementary.pdf]

Table S1. The reliability test of five THLQ subindices.

Indices	Items	Cronbach's Alpha	
		Validation study	Present study
General HL	42	0.9411	0.9456
(1) Accessing Information Relevant to Health	8	0.8975	0.9542
(2) Understanding Information Relevant to Health	7	0.8605	0.8495
(3) Questioning Information Relevant to Health	12	0.9023	0.9115
(4) Appraising Information Relevant to Health	7	0.8843	0.8248
(5) Applying Information to Change Behaviors	8	0.8214	0.7562

HL, Health literacy; THIQ, Thai health literacy questionnaire.

Table S2. Frequency and percentage of health literacy levels in the sample population.

Items (*n*=1,510)		Health Literacy Level, *n* (%)				
		Never/ Cannot Do	Very difficult	Difficult	Easy	Very Easy
**Accessing Information Relevant to Health**						
S1	What to do if someone is unconscious	779 (51.6)	37 (2.4)	104 (6.9)	398 (26.4)	192 (12.7)
S2	Symptoms require immediate hospitalization	518 (34.3)	46 (3.1)	109 (7.2)	654 (43.3)	183 (12.1)
S3	Health check-up or vaccinations should be given	520 (34.4)	48 (3.2)	122 (8.1)	658 (43.6)	161 (10.7)
S4	Self-prevention from communicable diseases	479 (31.7)	35 (2.3)	95 (6.3)	712 (47.2)	189 (12.5)
S5	How to maintain health	404 (26.8)	32 (2.1)	68 (4.5)	698 (46.2)	308 (20.4)
S6	Proper way to manage stress	434 (28.7)	33 (2.2)	91 (6.0)	768 (50.9)	184 (12.2)
S7	Reliable information about medicines, cosmetics, herbs and supplements	524 (34.7)	43 (2.9)	162 (10.7)	625 (41.4)	156 (10.3)
S8	Reliable information about health services, new products or equipment	645 (42.7)	106 (7.0)	223 (14.8)	433 (28.7)	103 (6.8)
**Understanding Information Relevant to Health**						
U9	Public media, online media or what to do in a medical emergency	175 (11.6)	45 (3.0)	158 (10.5)	874 (57.9)	257 (17.0)
U10	Why you should get a health screening	103 (6.8)	23 (1.5)	108 (7.2)	1060 (70.2)	216 (14.3)
U11	Understand why you need a vaccine	98 (6.5)	18 (1.2)	78 (5.2)	1042 (69.0)	274 (18.2)

U12	Warnings about how important and necessary healthy behaviors are	48 (3.2)	22 (1.4)	36 (2.4)	1016 (67.3)	388 (25.7)
U13	How to maintain good mental health	77 (5.1)	25 (1.7)	61 (4.0)	1073 (71.1)	274 (18.2
U14	Understand the labels for drugs, cosmetics, dietary supplements, herbs, medical devices and dangerous substances	132 (8.8)	47 (3.1)	167 (11.1)	872 (57.7)	292 (19.3)
U15	Description of new health services, products or equipment	334 (22.2)	105 (7.0)	280 (18.5)	653 (43.2)	138 (9.1)
**Questioning Information Relevant to Health**						
Q16	Ask your doctor about your rights, expenses and concerns	69 (4.6)	19 (1.3)	400 (26.5)	727 (48.1)	295 (19.5)
Q17	Ask the nurse about your rights, expenses and concerns.	71 (4.7)	22 (1.5)	461 (30.5)	710 (47.0)	246 (16.3)
Q18	Ask the hospital worker about your rights, expenses and concerns.	67 (4.5)	15 (1.0)	616 (40.8)	606 (40.1)	206 (13.6)
Q19	Ask your doctor about the disease and the practice of prevention	64 (4.3)	13 (0.9)	431 (28.5)	722 (47.8)	280 (18.5)
Q20	Ask the nurse about the disease and the practice of prevention	66 (4.4)	14 (0.9)	488 (32.3)	723 (47.9)	219 (14.5)
Q21	Ask the health volunteer about the disease and the practice of prevention	74 (4.8)	10 (0.7)	627 (41.5)	612 (40.5)	189 (12.5)
Q22	Ask your doctor for information on how to stay healthy	146 (9.7)	14 (0.9)	646 (42.8)	532 (35.2)	172 (11.4)
Q23	Ask the nurse for information on how to stay healthy	147 (9.7)	15 (1.0)	727 (48.2)	499 (33.0)	122 (8.1)
Q24	Ask the health volunteer for information on how to stay healthy	141 (9.3)	9 (0.6)	786 (52.1)	456 (30.2)	118 (7.8)
Q25	Ask your doctor about health products	167 (11.1)	17 (1.1)	669 (44.3)	514 (34.0)	143 (9.5)
Q26	Ask the nurse about health products	171 (11.3)	16 (1.1)	673 (44.6)	523 (34.6)	127 (8.4)
Q27	Ask the health volunteer about health products	180 (11.9)	12 (0.8)	645 (42.7)	522 (34.6)	151 (10.0)
**Appraising Information Relevant to Health**						
D28	Decide how you follow an emergency information	101 (6.7)	34 (2.2)	140 (9.3)	930 (61.6)	305 (20.2)

D29	Decide how you follow a complete treatment plan	56 (3.7)	24 (1.6)	87 (5.8)	1030 (68.2)	313 (20.7)
D30	Decide what you will do in order to use the drug correctly	78 (5.2)	20 (1.3)	97 (6.4)	1048 (69.4)	267 (17.7)
D31	Decide how you should exercise	52 (3.4)	21 (1.4)	101 (6.7)	1084 (71.8)	252 (16.7)
D32	Decide what foods to buy or how to cook	242 (16.0)	48 (3.2)	176 (11.7)	869 (57.5)	175 (11.6)
D33	Decide what activities you should do to reduce stress	56 (3.7)	15 (1.0)	52 (3.4)	1050 (69.6)	337 (22.3)
D34	Decide to choose a supplement, nutrition or herbs	121 (8.0)	81 (5.4)	223 (14.8)	919 (60.9)	166 (11.0)
**Applying Information to Change Behaviors**						
B35	Choose bland food more than sweet, salty, and oily food	24 (1.6)	37 (2.5)	286 (18.9)	871 (57.7)	292 (19.3)
B36	Do not share a spoon with others while eating	164 (10.9)	23 (1.5)	150 (9.9)	805 (53.3)	368 (24.4)
B37	Exercise to strengthen muscles 3-4 days a week.	209 (13.8)	40 (2.7)	276 (18.3)	724 (47.9)	261 (17.3)
B38	Manage your stress appropriately	38 (2.5)	18 (1.2)	110 (7.3)	1072 (71.0)	272 (18.0)
B39	Read and review the information provided with cosmetics, supplements or herbs	236 (15.6)	63 (4.2)	178 (11.8)	802 (53.2)	230 (15.2)
B40	Inspect health products, services, cosmetics, medical devices and hazardous substances	291 (19.3)	132 (8.7)	289 (19.2)	629 (41.7)	168 (11.1)
B41	If you find someone drowning, help by "yelling, throwing, handing" and calling 1669.	121 (8.0)	85 (5.6)	291 (19.3)	826 (54.7)	187 (12.4)
B42	Encourage all children to receive developmental assessment and vaccination as needed	75 (5.0)	30 (2.0)	94 (6.2)	882 (58.4)	429 (28.4)
